# Can vascular mortality be reliably ascertained from the underlying cause of death recorded on a medical death certificate? Evidence from 2800 adjudicated heart protection study (HPS) deaths

**DOI:** 10.1186/1745-6215-16-S2-P61

**Published:** 2015-11-16

**Authors:** William Herrington, Karl Wallendszus, Louise Bowman, Martin Landray, Jane Armitage

**Affiliations:** 1 CTSU, Nuffield Department of Population Health, University of Oxford, Oxford, UK

## Background

The validity of certified Underlying Cause of Death (UCD) for trial follow-up is unknown.

## Methods

HPS(1994-2001) flagged participants with UK national mortality registers and collected additional information for clinician adjudication of all reported deaths. Agreement between adjudicated (the gold standard) and certified UCD was assessed by Kappa(ĸ) statistics.

## Results

Of 20536 participants, 2835 died during trial follow-up, of whom 2778 (98%) were recorded by UK mortality registers.

Among these registered deaths, 1260 were adjudicated to coronary heart disease(CHD). Certified UCD agreed with 1152 of these, attributed 108 adjudicated CHD deaths to other causes (including 71 non-vascular causes) and wrongly ascribed 81 non-CHD deaths to CHD (ĸ=0.86, 95% confidence interval 0.84-0.88).

Of the 214 adjudicated stroke deaths, certified UCD agreed with 161 but attributed 53 to other causes and ascribed 26 non-stroke deaths to stroke (ĸ=0.78, 0.74-0.83). Of the 60 ischaemic stroke deaths, certified UCD recorded 7 as ischaemic stroke, 29 as unknown stroke and 24 to other causes, resulting in poor agreement (ĸ=0.19, 0.07-0.31). Overall, however, agreement for any vascular death remained good (ĸ=0.84, 0.82-0.86).

In HPS allocation to active treatment reduced vascular deaths by 17% (781/10269(7.6%) versus 937/10267(9.1%); risk ratio 0.83, 0.75-0.91). Had follow-up solely relied on certified UCD this result would have been materially unchanged (737(7.2%) versus 900(8.8%); risk ratio 0.81, 0.74-0.90).

## Conclusions

Certified UCD from UK mortality registers appears sufficiently reliable to ascertain vascular deaths, including CHD and stroke deaths considered separately, but additional information is required should the need to differentiate stroke subtypes arise.

